# A Foley Fallacy: A Case of Bladder Rupture after “Routine” Foley Catheter Placement

**DOI:** 10.1155/2018/7978126

**Published:** 2018-12-13

**Authors:** Shiva Poola, Arjun Mohan

**Affiliations:** Department of Internal Medicine, East Carolina University Brody School of Medicine/Vidant Medical Center, Greenville, NC, USA

## Abstract

Bladder injury is a rare condition mostly due to high-energy trauma. Bladder injury tends to be suspected during traumatic events in the setting of hematuria, pain, and voiding difficulty. Unfortunately, in end-stage renal disease patients who are oliguric or anuric these classic clinical findings would not be seen. We report a case of bladder rupture without a history of trauma or without a history of hematuria or voiding difficulties. To our knowledge this is the first case to describe such an injury with a lack of trauma history.

## 1. Introduction

Bladdery injury occurs due to blunt trauma causing disruption of the bladder and seen in approximately 1.6% of abdominal trauma. These injuries are found in high-energy blunt trauma involving the bony pelvis such as in motor vehicle collisions, falls, crush injuries, and blows to the lower abdomen [1]. On the contrary, there have been reports that bladder injury may occur with the lack of traumatic injuries. These cases have been associated with carcinoma, chronic cystitis, chronic catheterization, and outflow obstruction [2]. There is a paucity of reported cases documenting Foley catheterization as cause of bladder rupture. We describe a case of a female who was diagnosed with urinary bladder rupture after Foley catheter placement.

## 2. Case

**Figure 1:**
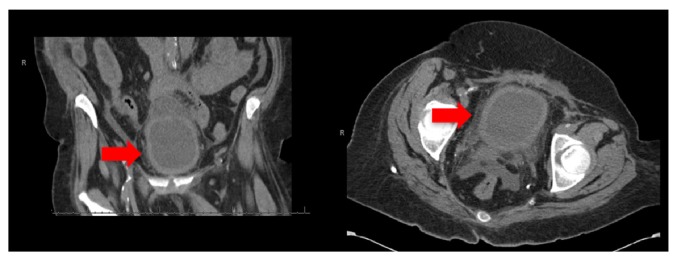
Distended bladder with thickened wall (arrow).

**Figure 2:**
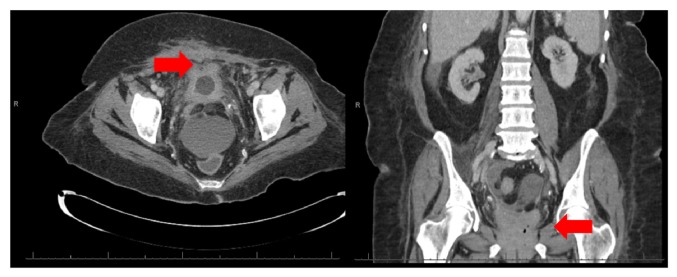
Extraperitoneal/intraperitoneal air with Foley catheter in place (arrow).

**Figure 3:**
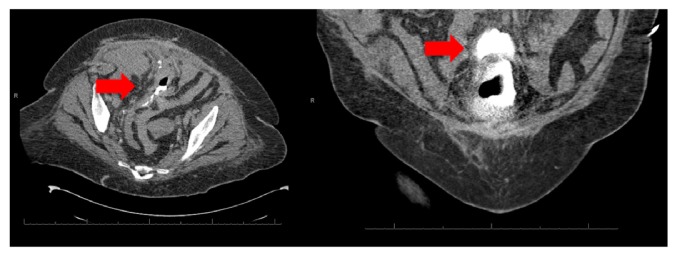
Contrast extravasation demonstrated with computed tomography cystogram (arrow).

A 62-year-old African American woman presented to the emergency department with complaints of abdominal pain, nausea, and vomiting after dialysis. Her history is significant for end-stage renal disease (ESRD) on chronic hemodialysis, morbid obesity, hypertension, type two diabetes, cerebellar vascular accident, and recent bilateral inguinal hernia repair. Her initial workup including computed tomography (CT) scan of her abdomen and pelvis was unremarkable outside of a distended bladder with thickened wall (Figure 1). Given these findings a Foley catheter was placed for bladder decompression. Over the next twenty-four hours she continued to have ongoing abdominal complaints including nausea and vomiting with worsening clinical picture concerning sepsis in the setting of progressive hypotension (systolic blood pressures in the 70s) and tachycardia (heart rate in the 100s). She had repeat computed tomography scan of her abdomen and pelvis without contrast which was concerning intraperitoneal and extraperitoneal air around the bladder with a Foley catheter in place (Figure 2). This prompted reevaluation of her bladder further with CT cystogram which demonstrated contrast extravasation (Figure 3). Within the next twenty-four hours the patient had an exploratory laparotomy which demonstrated pus in the pelvis, necrotic areas of the bladder, and an easily palpated Foley balloon. The patient underwent a partial cystectomy with primary repair. The patient had a complicated postoperative course that included a prolonged intensive care unit stay, postoperative hemorrhage, respiratory failure, sepsis, and pulseless electrical activity cardiac arrest. Given her complicated course the palliative care team was consulted and after multiple family meetings the decision was to pursue comfort care. The patient died shortly after while in hospice care in the palliative care unit.

## 3. Discussion

Bladder injury is a condition most likely due to high impact trauma. However spontaneous bladder rupture has been reported in several case reports without a history of traumatic event [3, 4]. Spontaneous bladder rupture has also been associated with malignant disease, outflow obstruction, or combined etiology [5, 6]. Studies have demonstrated bladder injury with bladder catheterization; however this has been demonstrated with chronic intermittent catheterization or chronic indwelling catheter placement as opposed to one time placement of a Foley catheter [7].

Matlock et al. conducted a 10-year review of trauma and found the incidence of bladder injury to be approximately 0.36 percent [8]. The American Urologic Association (AUA) most recent urotrauma guidelines from 2017 stated bladder injuries occurred in approximately 1.6% of blunt abdominal trauma [9]. Reports of spontaneous rupture have an incidence of 1:126,000 [10]. Although no study has demonstrated the incidence of catheter associated bladder rupture or injuries there are multiple studies which have identified catheter as the likely cause of bladder injury [7, 11–13]. While bladder injuries are rare, the overall mortality of bladder injuries can be as high as 34% [14].

In this report we describe a case of bladder rupture without a traumatic event or clinical history of hematuria, voiding difficulties, or malignancy. The patient in this scenario had a clinical history of ESRD and was known to be oliguric. During her extensive evaluation she had a distended bladder and therefore for decompression and sepsis workup had a Foley catheter placed. Given ongoing clinical deterioration she was found to have a bladder rupture on reimaging, which was thought to be secondary to Foley catheter placement.

Foley catheter associated bladder injury is rare but should be suspected in cases similar to ours. This patient's oliguric state may have led to delay in diagnosis. When suspected it was appropriately confirmed by a CT cystogram. The AUA has stated that to diagnose bladder injury a retrograde cystography is the technique of choice, either plain film or CT cystography [9]. There is no data published regarding the best diagnostic modality for bladder injury in renal failure or oliguric patients; however Matsumura et al. did report a case of renal failure with bladder rupture which was diagnosed with CT imaging [15]. In conclusion, intraperitoneal bladder rupture is difficult to assess and should be on the differential when evaluating deteriorating septic patients after Foley catheter placement, even without a history of trauma or without changes in urination.
